# Molecular Techniques and Ecological Data for Taxonomically Difficult Groups: A Case Study of a Morphologically Variable New Species in the Genus *Chrysobothris* (Coleoptera: Buprestidae)

**DOI:** 10.3390/insects17030291

**Published:** 2026-03-06

**Authors:** Botao Huang, Long Wu, Tao Ni, Rongxiang Su, Haitian Song, Rong Wang

**Affiliations:** 1 Fujian Academy of Forestry, Fuzhou 350012, China; hbotao623@foxmail.com; 2 State Key Laboratory of Marine Environmental Science, Key Laboratory of the Ministry of Education for Coastal and Wetland Ecosystems, College of the Environment and Ecology, Xiamen University, Xiamen 361102, China; long.w@foxmail.com; 3 State Key Laboratory of Agricultural and Forestry Biosecurity, College of Forestry, Fujian Agriculture and Forestry University, Fuzhou 350002, China; nitao17173@126.com; 4 Furong Institute of Biology (Xiamen) Co., Ltd., Xiamen 361004, China; surongxiang2022@126.com

**Keywords:** China, *Chrysobothris*, COI, jewel beetle, new species, taxonomy

## Abstract

Morphological characters of beetles can differ greatly, even within a single species, necessitating the integration of molecular techniques and ecological data for accurate taxonomical delineation, particularly within taxonomically challenging groups. *Chrysobothris*, a world-distributed genus of considerable size with a homonymy rate exceeding 1/5, frequently presents ambiguities in species boundaries. In this research, a series of *Chrysobothris* specimens collected from southern China were segregated into four sharply contrasting external morphotypes. A taxonomic ambiguity was initially posed: whether they represented several species, intraspecific polymorphism within a single species, or geographic/intraspecific variants of the similar species *Chrysobothris violacea* Kerremans, 1892. COI barcoding and phylogenetic analyses supported the conspecificity of these morphotypes and confirmed their distinction from *C. violacea* at the species level. Based on integrated evidence, we describe these specimens as *Chrysobothris borealina* Huang, Wu & Song, sp. nov., provide diagnostic characters with illustrations, and compare the new species with *C. violacea*. The species occurs in mid- to high-elevation pine and pine–broadleaf mixed forests and differs from *C. violacea* in both elevational range and phenology, indicating potential ecological differentiation. Additionally, we document a rare instance of a nymphal parasitengone mite (cf. Erythraeidae) attached to one female specimen.

## Simple Summary

Morphological characters of beetles can exhibit considerable variation, even within a single species. Within a *Chrysobothris* group from southern China, four morphotypes were suspected, raising the possibility that they represented multiple species or subspecies. By using COI barcode sequencing, we determined that all forms belong to the same species, which is genetically distinct from its closest relative species, *C. violacea* Kerremans, 1892. Herein, we describe and illustrate a new species, *Chrysobothris borealina* Huang, Wu & Song, sp. nov. Key morphological characters are summarized, ecological notes are provided, and the presence of mite attachment on one female specimen is reported.

## 1. Introduction

As integral components of forest ecosystems, buprestids are often employed as informative indicators in assessments of biodiversity and forest health [1]. However, morphological characters alone can be misleading in taxa exhibiting high variability, prompting increased reliance on molecular reassessments, and it often occurs in this family. An example of this could be that by using a combined analysis of COI and 16S ribosomal RNA sequences, complemented by morphological examination, the subspecies *Chrysochroa fulgidissima alternans* (Waterhouse, 1888) was elevated to species rank, and two new species were formally described from the *Chrysochroa fulgidissima* complex [2].

*Chrysobothris* Eschscholtz, 1829 is a nearly cosmopolitan genus distributed across most major zoo-geographical regions [3,4], and currently includes more than 700 described species [3]. According to Bellamy’s compilation within The World of Jewel Beetles (Cerambycids.com), the genus currently comprises 708 valid species, alongside 193 synonymic names [5]. This high degree of synonymy underscores the considerable taxonomic challenges within the group, where pronounced intraspecific variation and polymorphism—particularly in coloration and maculation—as well as potential morphological convergence, often complicate the delineation of species boundaries based solely on morphology [5,6].

Morphologically, species of *Chrysobothris* are often characterized by a serrate lateral margin of the elytra, at least in the posterior half or subapical region, frequently accompanied by small teeth or spines along the apical margin [7]. Traditional taxonomic diagnoses also emphasize features such as the sculpture of the pronotal disc, the form of the prosternum, and the structure of the ultimate abdominal ventrite in distinguishing *Chrysobothris* species [8]. In most *Chrysobothris* species, the shape of the apex of the last ventrite in males is much more deeply concave inward than in females, and in certain species groups, the apical margin of the last male ventrite bears a pair of spines [9]. Although a sexual color dimorphism is generally uncommon within the genus, extreme cases have occurred. A classic example is *Chrysobothris humilis* Horn, 1886, in which females exhibit a bright metallic green coloration while males are black and red [10].

In taxonomically challenging groups, such as *Chrysobothris*, extensive intraspecific variation, sexual dimorphism, and morphological convergence often obscure species boundaries, thereby complicating morphology-based identifications. Molecular techniques, particularly DNA barcoding based on the cytochrome c oxidase subunit I (COI) gene, provide an independent and objective means of testing whether divergent morphotypes represent distinct species or phenotypic variants within a single lineage [11]. In genera characterized by high morphological plasticity, such as *Chrysobothris*, molecular data are valuable for preventing erroneous species delineation or misidentification. Consequently, integrative taxonomic approaches that combine morphological and molecular evidence are essential for stabilizing species hypotheses within Buprestidae.

In East Asia, the genus *Chrysobothris* has attracted considerable taxonomic interest. For example, Kurosawa described several new species from China, Korea, and Japan, highlighting the species richness and morphological diversity of the genus within the transitional zone between the Oriental and Palaearctic regions [12]. Nevertheless, compared to the relatively comprehensive regional revisions conducted in North America and the Neotropical region, taxonomic studies on *Chrysobothris* in East Asia remain fragmented; most studies comprise descriptions of locally restricted new species or revisions of particular species groups from the Oriental region and adjacent island faunas [3,12,13]. In the genus *Chrysobothris*, some closely related species exhibit overlapping distributions and present two contrasting morphological patterns: external morphological convergence coupled with genital divergence, and genital conservatism alongside significant external morphological differentiation. Such inconsistencies pose challenges for species delineation based on a single character system.

Recent surveys conducted in tropical monsoon forests and secondary forests across southern China and neighboring areas continue to yield new species of Buprestidae, particularly within *Chrysobothris*, indicating that the taxonomy and faunal knowledge of the group in this region remains incomplete and retains considerable potential for refinement. In Indochina and adjacent areas, the discovery of new *Chrysobothris* taxa is still ongoing; for example, Hołyński (2020) described *Chrysobothris* (s. str.) *biplaga* from Cambodia [14]. Along with recent taxonomic work from Southeast Asia and nearby islands (e.g., [15]; see also the annual literature and new-taxon compilations in *BUPRESTIS*), a conservative tally suggests that ca. 20 *Chrysobothris* species have been newly described from Asia since 2000, providing an explicit regional backdrop for the present long-awaited addition from China [15,16]. To date, 24 species of *Chrysobothris* have been recorded in China [17].

Within the genus *Chrysobothris*, intraspecific polymorphism in coloration and markings is prevalent, yet its evolutionary underpinning remains inadequately elucidated. Here, we describe a novel and remarkably polymorphic species, *Chrysobothris borealina* sp. nov., from southern China, based on COI phylogenetic analyses and integrated morphological evidence. We also briefly discuss the potential adaptive significance of its color-pattern variation in montane coniferous and mixed coniferous–broadleaf forests ecosystems.

## 2. Materials and Methods

### 2.1. Insect Collection and Image Processing

A series of *Chrysobothris* specimens representing four markedly distinct external morphotypes in both sexes was examined. Due to the considerable variability in external coloration and maculation within the genus, these morphotypes were treated a priori as unresolved taxonomic units requiring further validation. Specifically, we aimed to determine whether the collected material constitutes: (i) a single polymorphic species, (ii) multiple species (potentially including one or two undescribed taxa), or (iii) geographic or intraspecific variants of the related species *Chrysobothris violacea.* Specimens were primarily collected through sweep-netting; additional specimens were obtained via hand-collecting, including individuals gathered from the ground and anthropogenic structures, such as railings.

The four morphotypes are characterized as follows:

Type 1: (male, typical morph, including Zhejiang, Hunan, Jiangxi, Chongqing, and Guangxi. Figure 3a,c).

This phenotype corresponds to the predominant appearance observed across the sample set. Males exhibit considerable consistency in overall habitus and dorsal coloration pattern, herein regarded as the “baseline” male morphotype.

Type 2: (female, Guangxi–Jiangxi predominant. Figure 5a).

Characterized by a pronotum bearing a large, relatively uniform blue–green patch covering most of the disc. This morphotype occurs predominantly in females, especially among specimens originating from Guangxi and Jiangxi provinces.

Type 3: (Hunan female, Figure 5b–Sichuan female, Figure 11d).

Displays a well-defined, vase-shaped purple macula situated centrally on the pronotal disc, which is largely uniform and devoid of greenish tones. This form was observed only in females until now.

Type 4: (Zhejiang morph. Figure 5c and Figure 11a,e).

Distinguished by the absence of typical green terminal maculation on the apical area of the elytra, the elytral apex is essentially immaculate with respect to green markings.

Specimen examination was conducted using a Keyence VHX-5000 digital microscope (Keyence Corporation, Tokyo, Japan) equipped with a VH-Z20R zoom lens (Keyence Corporation, Tokyo, Japan). Images were captured using focus-stacked techniques, and the resultant image layers were combined into plates using Adobe Photoshop CC 2018 (Adobe Systems Inc., San Jose, CA, USA). Morphometric measurements were conducted according to methodologies outlined in references [18,19], defined as follows:

Body length: The distance from the apex of the head to the apex of the elytra.

Body width: The maximum width of the body, typically at the humeri.

Aedeagus length: The distance from the base to the apex of the aedeagus.

Aedeagus width: The maximum width of the parameres.

All specimens examined in this study are deposited in the following six collections:

BMNH: The Natural History Museum (British Museum of Natural History), London, UK;

CHH: Private Collection of Hao Huang, Qingdao, China;

CHTS: Private Collection of Hai-Tian Song, Fuzhou, China;

CTH: Private Collection of Takaharu Hattori, Yokohama, Japan;

FAF: Fujian Academy of Forestry, Fuzhou, China;

SYSBM: Sut-Yat Sen University Biomuseum, Guangzhou, China.

Holotype is deposited in FAF and paratypes in SYSBM, CHTS, CHH and CTH. For details, please check the section “**Type materials**”.

### 2.2. DNA Extraction and Sequence Analysis

Genomics DNA was extracted using TIANamp Micro DNA Kit (DP316, Tiangen, Beijing, China), following the manufacturer’s protocol. Target regions were amplified via polymerase chain reaction (PCR) using the primers listed in Table 1. PCR products were purified and subjected to Sanger sequencing by Biosun Co., Ltd. (Fuzhou, China) to obtain the cox1 sequences.

The degree of substitution saturation was assessed using DAMBE (version 7.3.32) by calculating the Iss and Iss.c critical values for the target sequences against all available Buprestidae sequences retrieved from NCBI. Sequence alignment and phylogenetic analysis were performed in MEGA (version 7.0). A maximum likelihood (ML) tree was constructed based on the Kimura 2-parameter genetic distance model. The resulting phylogenetic tree was visualized and annotated using the Interactive Tree of Life online tool iTOL (https://itol.embl.de/ (accessed on 1 February 2026)).

Sample code abbreviations. To ensure concise, consistent referencing of the sequenced individuals throughout the text, tables, and phylogenetic figures, we assigned each voucher a standardized specimen code. Initially, these coded specimens appeared strikingly similar in overall habitus, body size, and key genital/external characters, exhibiting partially overlapping external variation. To minimize potential preconceived assignments based solely on traditional morphology and guarantee equivalent treatment for all candidate forms, we processed every coded individual using an identical DNA extraction, PCR amplification, and Sanger sequencing workflow. Each code combines the Chinese province of origin + sex + (optional) individual number: the first two letters denote the Chinese province (ZJ for Zhejiang; GX for Guangxi; HN for Hunan; YN for Yunnan; and JX for Jiangxi), the third letter signifies sex (M for male and F for female), and a two-digit suffix (01 and 02) is appended when multiple individuals share the same province–sex combination (GXF01 and GXF02). These codes are used solely as shorthand labels to link each sequenced specimen unambiguously to its voucher specimen and sequence data (and, where applicable, GenBank accession numbers), thereby improving traceability and reproducibility while avoiding overly long labels in figures and tables. A mapping between the original molecular numbers and the newly assigned specimen codes is provided in [3.2 Type materials].

## 3. Results

### 3.1. Molecular Results

#### 3.1.1. DAMBE Analysis

Evolutionary analysis of the sequence was performed using DAMBE v7.3.32. The calculated ISS index of 0.2329 was significantly lower than the critical value ISS.C = 0.8003 (*p* < 0.01) for the LCO-HCO primer pair, and the ISS value of 0.0315 was also significantly lower than the ISS.C = 0.7880 (*p* < 0.01) for the JERRY-PAT primer pair. These results indicate negligible substitution saturation, confirming the suitability of these sequences for robust phylogenetic inference.

#### 3.1.2. Genetic Analysis of LCO-HCO Primer Group

Genetic distances among the 15 sequences were estimated under the Kimura 2-parameter model with bootstrap testing (1000 replicates). As shown in Table 2, the minimum genetic distance (0.001) was observed between samples HNF and HNM, and the genetic distances among samples ZJM, GXF01, ZJF, HNF, and HNM were all lower than 0.022, indicating a close phylogenetic relationship. In contrast, genetic distances between these five taxa and the remaining ingroup species exceed 1.234, reflecting considerable phylogenetic divergence.

**Figure 1:**
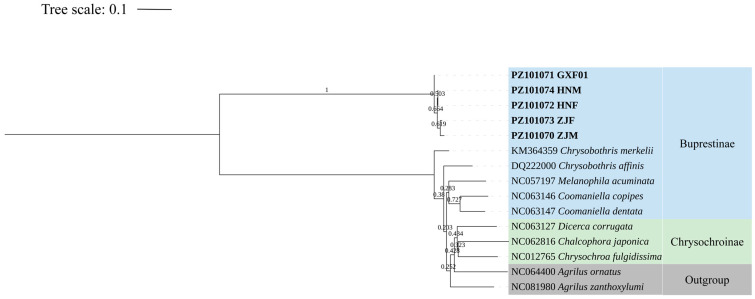
Maximum likelihood tree based on the analysis of COI gene sequences of samples ZJM, ZJF, HNM, HNF, GXF01 (later identified as *Chrysobothris borealina* sp. nov.) and other Buprestinae and Chrysochroinae species. Outgroup: Agrilinae (*Agrilus ornatus* and *Agrilus zanthoxylumi*). Rooted at the middle point. Bold branches indicate the species sequenced at this time.

A maximum likelihood phylogeny was reconstructed under the Kimura 2-parameter model, with branch support evaluated through 1000 bootstrap replicates. The phylogenetic tree revealed three major branch clusters, each with bootstrap confidence values exceeding 95%. A distinct and well-supported cluster comprising the five newly sequenced samples was evident, characterized by significant branch lengths separating them from other ingroup taxa (Figure 1). Among the compared groups, specimens from the subfamily Buprestinae exhibited the closest phylogenetic relationship to the five samples.

#### 3.1.3. Genetic Analysis of Primer JERRY–PAT Group

In the genetic analysis of the JERRY–PAT group, employing the same model as the LCO-HCO group (Table 3), the intragroup pairwise genetic distances ranged from 0.01 to 0.232. The genetic distances for samples ZJF, GXF02, and JXM reached the minimum value of 0.01. Sample HNF exhibited genetic distances ranging from 0.013 to 0.016 relative to ZJF, GXF02, and JXM. Additionally, the genetic distances between samples YNM and samples HNF, ZJF, GXF02, and JXM ranged from 0.067 to 0.075. Among the remaining ingroup taxa, the two species of genus *Chrysobothris* showed the closest genetic distance to those five samples, with genetic distances varying between 0.116 and 0.135.

**Figure 2:**
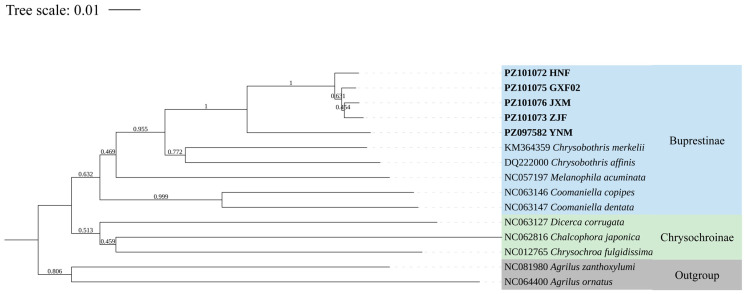
Maximum likelihood tree based on the analysis of the cox1 gene sequences of samples ZJF, HNF, GXF02, JXM (later all identified as *Chrysobothris borealina* sp. nov.), YNM (*C. violacea*), and other Buprestinae and Chrysochroinae species. Outgroup: Agrilinae (*Agrilus ornatus* and *Agrilus zanthoxylumi*). Rooted at the middle point. Bold branches indicate the species sequenced at this time.

Using the same methods as applied to the LCO-HCO group, the phylogenetic reconstruction (Figure 2) revealed that samples HNF, ZJF, GXF02, JXM, and YNM formed a well-supported clade, with bootstrap values exceeding 90%. Within this clade, samples HNF, ZJF, GXF02, and JXM exhibited shorter branch lengths and smaller genetic divergence values compared to YNM, indicating a closer evolutionary relationship among them. In contrast, conspecific specimens within the same genus or subfamilies display bootstrap values approaching 100%, consistent with closer genetic affiliations. Both the K2P genetic distance metrics and the phylogenetic relationships clearly demonstrate that species ZJF, HNF, GXF02, JXM, and YNM are not conspecific.

### 3.2. Taxonomy

Family Buprestidae Leach, 1815.

Subfamily Buprestinae Leach, 1815.

Tribe Chrysobothrini Gory & Laporte, 1837.

Genus *Chrysobothris* Eschscholtz, 1829.

Subgenus *Chrysobothris* Eschscholtz, 1829.

Type species: *Buprestis chrysostigma* Linnaeus, 1758.

***Chrysobothris borealina* Huang, Wu & Song, sp. nov.**

Chinese common name: 极光星吉丁

urn:lsid:zoobank.org:pub:3D2D9390-378E-4B49-A9BF-23D2661DB973

**Type locality**. China, Zhejiang Province, Lishui City, Baishanzu National Park, 27°44′35″ N, 119°11′12″ E, 1200 m.

**Figure 3:**
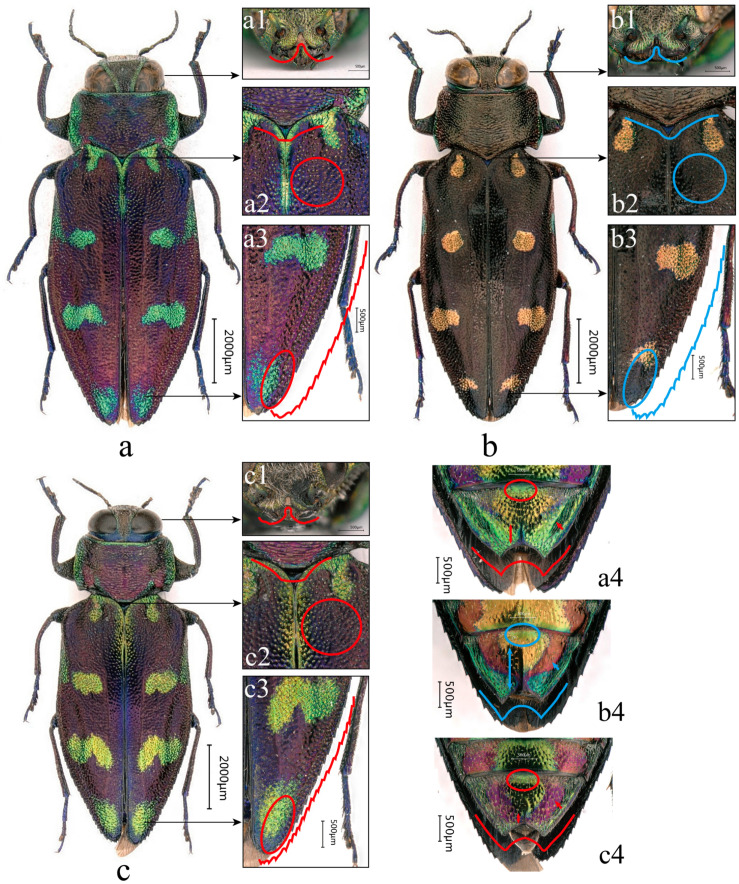
Adult habitus and selected diagnostic characters of males. (**a**) *C. borealina* sp. nov., ♂ holotype from Zhejiang, ZJM, Type 1; (**b**) *C. violacea*, ♂ from Laos, LAM; and (**c**) *C. borealina* sp. nov., ♂ paratype from Hunan, HNM, Type 1. (**a1**–**c1**) Clypeus in frontal view. (**a2**–**c2**) Posterior margin of pronotum and basal part of elytra. (**a3**–**c3**) Apicolateral region of elytra. (**a4**–**c4**) Apex of abdomen in ventral view. Red markings indicate comparative characters of *C. borealina* sp. nov.; blue markings indicate comparative characters of *C. violacea*. Scale bars as indicated.

**Type materials:**

**Figure 4:**
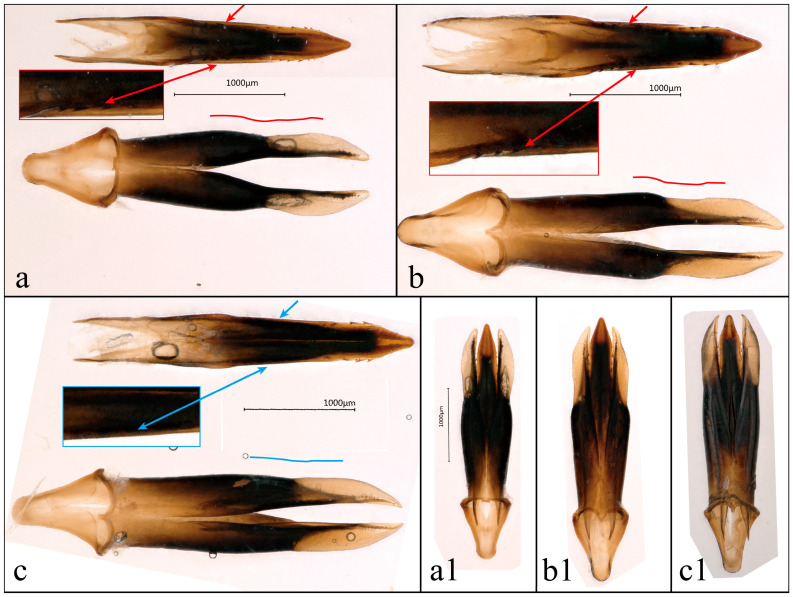
Aedeagus of *Chrysobothris borealina* **sp. nov.** and *C. violacea* Kerremans, 1892. (**a**) *C. borealina* **sp. nov.**, ♂ holotype from Zhejiang, ZJM, Type 1; (**b**) *C. borealina* **sp. nov.**, ♂ paratype from Hunan, HNM, Type 1; and (**c**) *C. violacea*, ♂ from Laos, LAM. In (**a**–**c**), upper images show the median lobe in dorsal view and lower images show the parameres in ventral view after separation. (**a1**–**c1**) Aedeagus in ventral view. Red markings indicate comparative characters of *C. borealina* **sp. nov.**; blue markings indicate comparative characters of *C. violacea*.

**Holotype:** ♂ (FAF): China, Zhejiang Province, Lishui City, Qingyuan County, Baishanzu National Park, 27°44′35″ N, 119°11′12″ E, alt. 1200 m, 25 July 2023, leg. Jing-Xiang Luo (Figure 3a, Figure 4a, Figure 8a and Figure 9a,i; molecular No. ZJM), Type 1.

**Figure 5:**
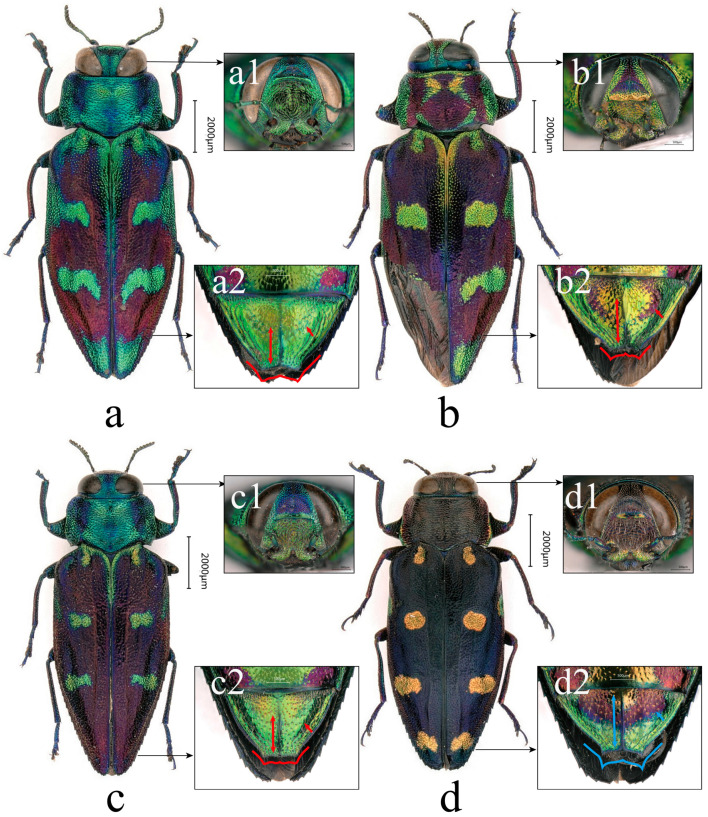
Female habitus and selected diagnostic characters of *Chrysobothris borealina* **sp. nov.** and *C. violacea* Kerremans, 1892. (**a**–**c**) *C. borealina* **sp. nov. paratypes:** (**a**) from Guangxi, GXF01, Type 2, (**b**) from Hunan, HNF, Type 3, (**c**) from Zhejiang, ZJF, Type 4, and (**d**) *C. violacea*, from Yunnan. (**a1**–**d1**) Head in frontal view. (**a2**–**d2**) Terminal abdominal ventrites in ventral view. Red markings indicate comparative characters of *C. borealina* sp. nov.; blue markings indicate comparative characters of *C. violacea*.

**Paratypes:** 1♀ (CHTS): China, Zhejiang Province, Quzhou City, Jiangshan City, Xianxialing, alt. 930 m, 28°19′13″ N, 118°39′41″ E, 21 Jun 2025, leg. Xuan-Xuan He (Figure 5c, Figure 7b, Figure 8f, Figure 9g,n,r and Figure 11e; molecular No. ZJF), Type 4; 1♂ (SYSBM, with the number EN-431711): China, Hunan Province, Zhangjiajie City, Tianmen Mountain National Forest Park, alt. 1300 m, 29°05′01″ N, 110°48′78″ E, 23 July 2023, leg. Ping Zhao (Figure 3c, Figure 4b, Figure 8b, Figure 9b,j and Figure 11b; molecular No. HNM), Type 1; 1♀ (CHTS): China, Hunan Province, Zhangjiajie City, Tianmen Mountain National Forest Park, alt. 1300 m, 29°05′01″ N, 110°48′78″ E, 18 July 2023, leg. Ping Zhao (Figure 5b, Figure 7d, Figure 8e and Figure 9f,m,q; molecular No. HNF), Type 3; 1♂ (CHTS): China, Jiangxi Province, Wugong Mountains, Wufa Inn, 27°29′30.84″ N, 114°12′07.99″ E, alt. 1566 m, 17 July 2024, leg. Jin-Qiao Zhu (molecular No. JXM), Type 1; 1♀ (CHTS): China, Jiangxi Province, Pingxiang City, Wugong Mountains, near Zhong’an Cableway, 27°27′29.12″ N, 114°10′19.98″ E, alt. 1342 m, 18 July 2024, leg. Jin-Qiao Zhu (molecular No. JXF), Type 2; 1♀ (CHH): China, Fujian Province, Wuyi Mountains, Dazhulan, 26°33′18.94″ N, 106°45′09.50″ E, alt. 875 m, 9 July 2009, leg. Yong-Xiang Wu, Type 2; 1♂ (CTH): China, Guangxi Zhuang Autonomous Region, Hezhou City, Yao Autonomous County, Mt. Caifu, 1-11 Jun 2002, leg. Jing-Ke Li, Type 1; 1♀ (FAF): China, Guangxi Zhuang Autonomous Region, Jinxiu Yao Autonomous County, alt. 1300 m, 1 July 2017, leg. Jin-Teng Zhao (Figure 5a, Figure 7a,c, Figure 8d, Figure 9e,i,p and Figure 11c; molecular No. GXF01), Type 2; 1♀ (SYSBM, with the number EN-431712): China, Guangxi Zhuang Autonomous Region, Laibin City, Jinxiu Yao Autonomous County, Dayaoshan Mountains, alt.1324 m, 24°06′36.75″ N, 110°11′02.54″ E, 20 Jun 2016, leg. Jin-Teng Zhao (molecular No. GXF02), Type 2; and 1♀ (CHTS): China, Chongqing Municipality, Jiangjin District, Mt. Chaqi (Chaqi Shan), 10 Aug 2010, alt. 1248 m, leg. Jian-Yue Qiu, Type 3.

**Description of the Holotype (**Figure 3a**):**

Body length 11.5 mm, body width 4.7 mm; aedeagus approximately 3.6 mm long and 0.85 mm wide. Body medium-sized, narrowly elongate–ovate, convex dorsally and ventrally; elytra widest at midlength, sharply narrowing towards apex.

**Head** short, transversely expanded in dorsal view, slightly narrower than anterior margin of pronotum, and moderately convex overall; outline of frons slightly projecting beyond general outline of head; and interocular distance relatively narrow. Head dorsally metallic blue–green to bluish violet, changing to bluish violet to violaceous with a weak sheen depending on angle of incident light. Frons short, conspicuously narrowed from top to bottom, disc slightly elevated; at fronto–vertexal boundary with a strong, slightly arcuate and weakly sinuate transverse carina extending across frons. Vertex behind transverse carina densely covered with coarse, umbilicate punctures. Frontal surface (in frontal view): area of frons anterior to transverse carina with distinct concentric transverse wrinkles, radiating in a wave-like pattern around a shallow central depression; areas near inner margins of compound eyes with dense umbilicate punctures combined with irregular reticulate wrinkles, many punctures coalescing to form irregular, polygonal, honeycomb-like cells. Median part of frons with a shallow longitudinal groove extending dorsad from between antennal insertions and still discernible within concentric wrinkles, often forming a darker, shallow depression in upper part, appearing inverted Y-shaped or fork-like in frontal view. Frons densely clothed with short to moderately long, pale yellowish to grayish white setae, mostly suberect to semi-appressed; setae particularly dense along inner margins of compound eyes and on lower margin of frons near clypeus, but sparser along median axis. Vertex relatively broad, slightly convex; sculpture similar to that of frons, but transverse wrinkles weaker, punctures slightly finer and more widely spaced; vertex with scattered longer, erect, pale brown to grayish white setae, most of them inclined anteriorly. Eyes large, oval, moderately and slightly bulging; ocular surface following outline of gena; inner margins smoothly curved. Gena short, moderately convergent ventrally, sculpture and pubescence similar to but slightly coarser than on frons. Clypeus short and transverse, nearly trapezoidal in frontal view, surface with fine dense punctures and sparse short setae; anterior margin with a deep median emargination forming two rounded, obtuse lobes, labrum partly exposed in emargination (light brown). Antennae inserted at about one-third of eye height below lower margin of eyes; antennal sockets relatively deep. Antennae short, not reaching posterior angles of pronotum, 11-segmented: scape moderately stout, subfusiform; pedicel short, nearly globular; antennomere 3 slightly longer; antennomeres 4–10 progressively wider and slightly expanded laterally, weakly serrate; terminal antennomere subovate. Basal antennomeres mostly metallic blue–green to coppery green, gradually becoming dark coppery green to dark brown towards apex, densely clothed with short, semi-appressed pale setae.

**Pronotum**. Disc with purple to purplish–brown metallic reflections; along anterior margin, lateral margins (and near basal margin) with distinct metallic blue–green reflections; golden–green reflective patch present near posterolateral angles. Anterior margin weakly produced; pronotum widest at base, anterior margin narrower than basal margin; sides moderately sinuate in middle. Surface densely sculptured with irregular punctation; transverse punctures on disc more strongly impressed.

**Scutellum** triangular, dark in color.

**Elytra**. Dorsally metallic purplish–blue to purplish–red, with strong iridescent luster, markedly wider than pronotum; humeral width ca. **1.4×** width across posterior angles of pronotum, elytra ca. **1.2×** as long as wide. Elytra evenly convex from base to apex; suture forming a low and narrow sutural costa; remainder of disc with a distinct longitudinal costa. Basal margin slightly constricted; humeral angles broadly rounded, humeral calli moderately elevated but not strongly produced laterally; basal margin strongly protruding anteriorly on each side of scutellum; basal and humeral depressions clearly defined. Disc punctation slightly finer and more even than on pronotum, densely covered with medium-sized round depressions, interspaces narrow and with a strong metallic sheen; purple ground-color areas lacking true striae. Sculpture of green macular areas conspicuously coarser, punctures expanded and fused into alveolate, honeycomb-like cells with slightly elevated margins, making maculae appear slightly depressed in dorsal view; a bluish–green transition zone present along macular boundaries. Elytra nearly glabrous, with extremely short and sparse pale setae along lateral and apical margins. Lateral margins from humeri to about midlength nearly straight or slightly outwardly arcuate, then rapidly converging toward apex; from about midlength to apex finely serrate with small triangular teeth, more distinct near subapical region and continuing almost to apical angle; apical outline finely serrate, without distinct apical spines. Each elytron with four principal bright green maculae: (1) basal macula on each side of scutellum forming a green sutural band in basal two fifths and extending outward toward humeri; (2) a subcircular to irregular macula near lateral margin at about midlength, connected to lateral green area; (3) a posteromedian wavy or vermiform band-like macula in outer half of posterior third, directed obliquely inwards and not reaching suture; and (4) a conspicuous apical macula, golden green, elliptical to subtriangular and cap-like, close to outer apical area and not attaining apical margin.

**Venter**. Ventral side glossy, with strong metallic luster. Thoracic venter predominantly emerald green; abdomen bright yellowish green to golden green, with purplish–blue to purplish–red metallic patches producing strong contrast. Thoracic venter and abdominal ventrites with medium-sized umbilicate depressions; punctures deep and dense, locally partially confluent into shallow alveolate cells; interspaces narrow, smooth and strongly reflective. Pubescence sparse on median areas (setae extremely short and scattered), but distinctly denser laterally: longer pale yellowish–white, semi-appressed to slightly erect, woolly setae forming tufts on lateral portions of thoracic sterna, around coxal cavities, and along lateral margins of ventrites. Hypomeron sculptured as on pronotal dorsum, with medium to slightly coarse, cell-like punctation. Prosternal process narrow between procoxae, posteriorly expanded into a triangular to wedge-shaped plate and then narrowing apicad; expanded portion subtrilobate with distinct lateral lobes, posteriorly prolonged into a slender acute process; surface densely punctate, with short appressed pale setae and irregular fine transverse wrinkles between some punctures; around coxal cavities with longer white woolly setae. Abdomen slightly convex; ventrites gradually narrower posteriorly, median line weakly elevated. Ventrites densely punctate with round to umbilicate depressions; punctures slightly finer and sparser in central yellowish–green to golden–green areas, but coarser and denser near lateral purple patches, locally confluent and tending to longitudinal alignment. Each ventrite with a pair of purplish–blue to purplish–red metallic patches near lateral margins, regularly repeated by segment and giving a banded appearance in ventral view. Median areas with extremely short sparse setae, setae somewhat concentrated near transverse sutures and around lateral purple patches. Longitudinal lateral callosities along ventral margins not developed. Anal ventrite large, subtriangular, broad at base and narrowed posteriorly; apical margin deeply emarginate medially, inner margin of emargination usually fringed with short dense setae. Sculpture similar to preceding ventrites but slightly finer; central area bright yellowish–green, often with faint purplish iridescence; short appressed pale setae denser than on middle of other ventrites, especially along lateral and apical margins; submarginal ridge distinct.

**Legs**. All legs somewhat robust overall; legs metallic blue–green to bluish–violet or purplish–copper, matching ventral coloration, tarsi mostly dark brown to dark coppery. Femora and tibiae with rather dense punctation and conspicuous setal pits, with strong metallic reflections; pubescence consisting mainly of pale setae: in addition to scattered short setae, femora and tibiae (especially along ventral margins and near joints) bearing more numerous, longer, semi-appressed to slightly erect pale setae. Femora rather thick, subfusiform to subclavate; fore and mid femora relatively robust, hind femora slightly slender and elongate. Surface of femora with punctures of medium density, setal pits conspicuous, bearing short setae interspersed with longer pale setae, more evident near bases and along ventral margins. Fore femur without obvious ventral tooth visible in available photographs. Tibiae relatively slender; protibiae nearly straight or only slightly curved, meso- and meta-tibiae gently arcuate outward; outer margin forming a weak carina and appearing slightly acute. Surface of tibiae with punctation sparser than on femora; in dorsal view, a fine, roughened band formed by setal pits and short setae is visible. Apices of tibiae slightly expanded and obliquely truncate; setae denser along apical margins and at joints. Tarsi 5-segmented, appearing “pseudotetramerous”: tarsomeres 1–3 comparatively conspicuous, tarsomere 4 markedly shorter and partially concealed beneath posterior margin of tarsomere 3, and tarsomere 5 slender, projecting beyond tarsal pads. Ventral surfaces of tarsomeres with well-developed pulvilli, facilitating adhesion to substrate; first metatarsomere relatively long, approximately equal to or slightly longer than combined length of tarsomeres 2 and 3. Claws paired, slender and curved, apices sharp; under available magnification, no obvious basal tooth or swelling is visible, thus claws can be regarded as simple.

**Aedeagus**. Overall slender and nearly symmetrical. In dorsal view, median lobe slender, slightly broadened at base; in basal half, lateral margins are almost parallel, then gradually narrowing from about middle towards apex. Apical one-third distinctly tapering, apex acute and slightly curved ventrad. Dorsal median area more darkly pigmented and strongly sclerotized, with relatively narrow lateral translucent areas. From about middle, outer margins are slightly arcuate outward, and along subapical outer margin there is a row of minute, spine-like denticles; these denticles are short and dense, giving subapical outer outline a distinctly finely serrulate appearance. In ventral view, median lobe narrowly fusiform, slightly constricted near middle and gradually tapering towards apex; boundary between median lobe and parameres clearly delimited. Parameres slightly shorter than median lobe; in ventral view slender and almost falcate. From base to middle, outer margin is distinctly expanded outward, being widest at about midlength, then markedly narrowed and curved inward; apices gradually tapered, slightly upturned dorsally, tips nearly parallel and only slightly separated. Inner margins from middle to apex shallowly concave; outer outline strongly arcuate, forming a distinctly bow-shaped curve. Pigmentation of parameres gradually darkening from paler base toward distal part, apices slightly translucent.

**Figure 6:**
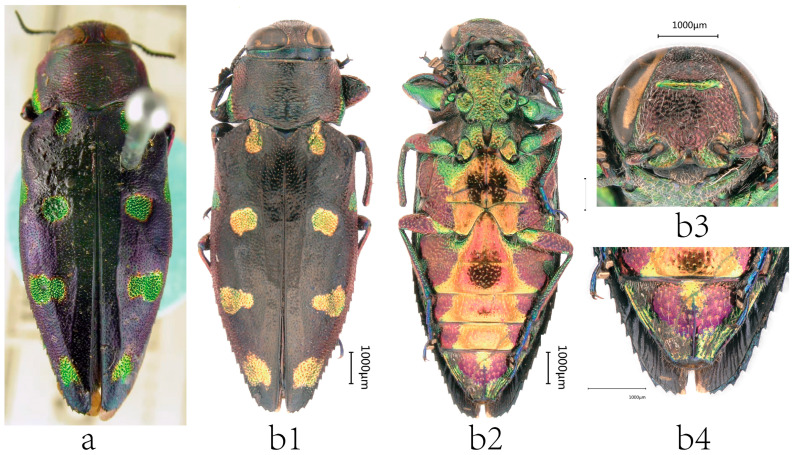
Variation in *Chrysobothris violacea* Kerremans, 1892. (**a**) Syntype specimen from Myanmar (deposited in BMNH, photo by Eduard Jendek, with permission). (**b1**–**b4**) Female specimen from Vietnam.

**Sexual dimorphism**. Females are overall larger, with the body more laterally expanded, whereas males are more narrowly elongate. Body length in males is approximately 10.3–11.9 mm, and in females is 12.4–13.9 mm. In the available series, females show a slightly higher mean L/W (≈2.66) than males (≈2.56), suggesting a generally more elongate habitus in females. On the dorsal surface, the golden–green reflective areas and maculae tend to be more extensive and brighter in females; in some individuals, the median and posterior elytral maculae more readily form transverse or wavy bands, see Figure 5a–c. In contrast, males more often show separated, discrete maculae with relatively well-defined margins (although considerable variation is present), see Figure 3a3,c3. In males, the apical margin of the anal ventrite is deeply emarginate, forming a broad U-shaped to nearly semicircular notch, see Figure 3a4,c4. In females, the apical emargination of the anal ventrite is shallower and often appears as a shallow double concavity; the apical opening may appear wider, and the female anal ventrite is generally more conspicuous than in males, see Figure 5a2–c2.

**Figure 7:**
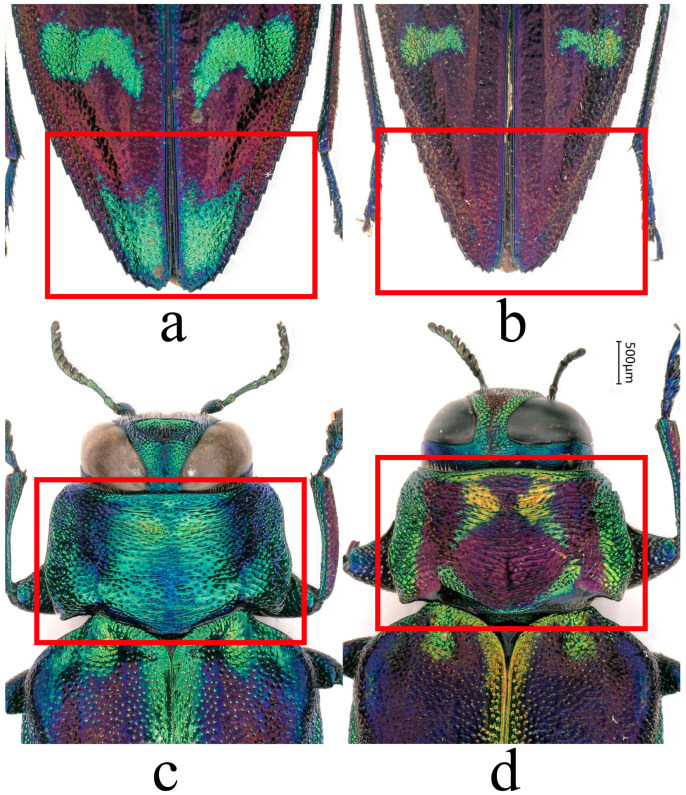
Intraspecific variation in the color pattern of *Chrysobothris borealina* **sp. nov.** (female specimens). (**a**,**b**) The elytral apex in dorsal view. (**c**,**d**) The pronotum in dorsal view. The red rectangles delimit the regions compared. (**a**,**c**) The paratype from Guangxi, (**b**) the paratype from Zhejiang, and (**d**) **the** paratype from Hunan.

**Variation**. The metallic ground color and colored maculation on the pronotum show considerable intraspecific variation. Some individuals exhibit largely continuous bluish–green metallic reflections on the disc with only weak purplish sheen (Figure 7c), whereas in other individuals, most of the pronotal disc is covered by purple–cupreous to metallic violet coloration, and the extent and shape of the green or yellowish–green patches vary greatly (Figure 7d). Elytral apical maculation is likewise highly variable: in some individuals, the subapical region bears a pair of well-delimited green to yellowish–green spots (Figure 7a), while in other specimens these apical maculae are markedly reduced or completely absent, leaving only the uniform metallic ground color at the apex (Figure 7b). The median emargination of the apical margin of the anal ventrite varies slightly among individuals.

**Etymology**. The species epithet *borealina* is derived from the Latin *borealis* (meaning “northern” or “of the north wind”, ultimately traceable to *Boreas*, the god of the north wind in Greek mythology). It alludes to the aurora-like impression produced by the external appearance of this new species: the elytra have a deep purple (cupreous violet) ground color overlaid with extremely vivid emerald green maculae. Under light, these maculae show a strong metallic iridescence and sharp contrast against the ground color, and their strongly variable patterns among individuals are reminiscent of the shifting luminous bands of the northern lights (*Aurora borealis*).

**Differential diagnosis**. The new species is similar to the common species *Chrysobothris violacea* Kerremans, 1892; their distinguishing characteristics can be seen in detail in Table 4.

**Figure 8:**
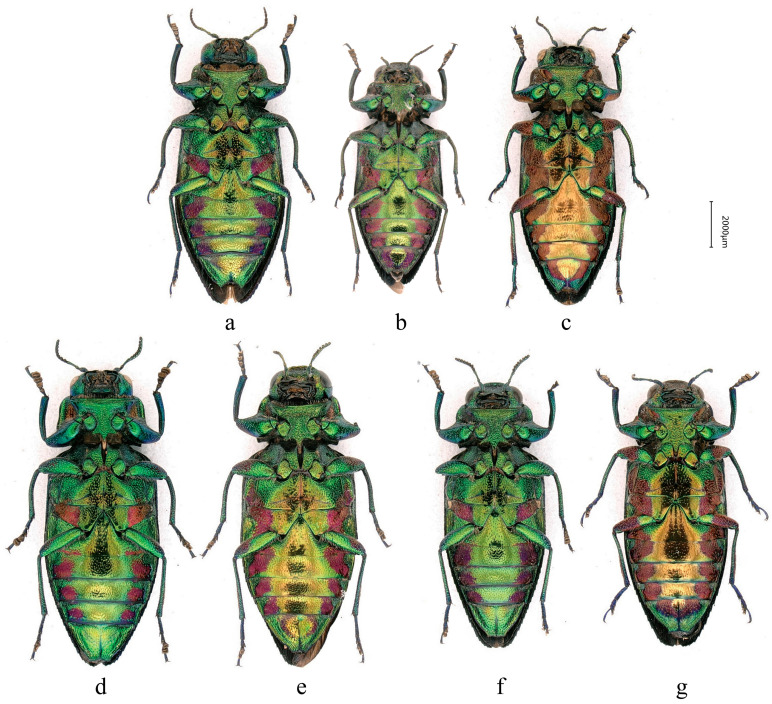
Ventral habitus of *Chrysobothris borealina* sp. nov. (**a**,**b**♂,**d**–**f**♀) and *C. violacea* Kerremans, 1892 (**c**♂,**g**♀). (**a**) Holotype from Zhejiang, (**b**) paratype from Henan, (**c**) from Laos, (**d**–**f**) paratypes from Guangxi, Hunan, and Zhejiang, and (**g**) from Yunnan.

**Figure 9:**
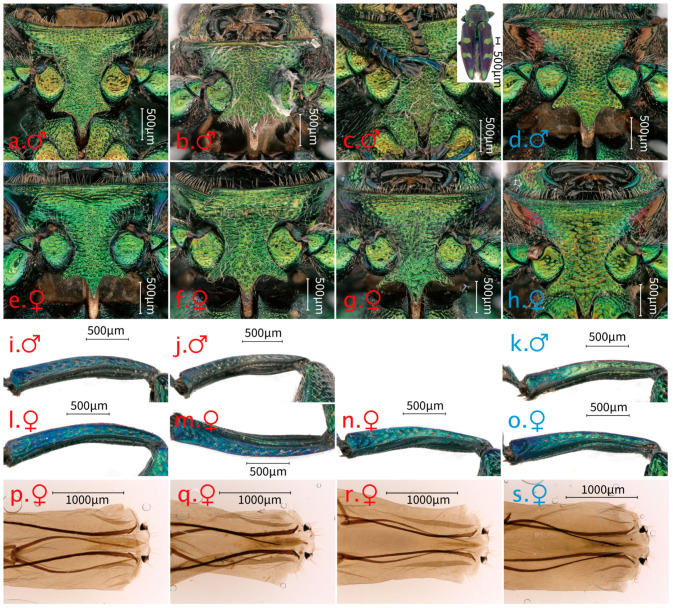
Shared morphological characters of *Chrysobothris borealina* sp. nov. and *C. violacea* Kerremans, 1892. The red labels indicate individuals of *C. borealina* sp. nov., and the blue labels indicate individuals of *C. violacea*. (**a**–**h**) The prosternal process and anterior ventral thoracic region of males and females of both species showing the similar overall shape of the prosternal process, sculpture, and punctation pattern. (**i**–**o**) The protibiae of males and females of both species in lateral view, illustrating the generally similar proportions, curvature, and armature of the fore tibia. (**p**–**s**) The female ovipositor in ventral view, demonstrating the similar structure and proportions of the ovipositor between the two species. Scale bars as indicated. (**a**,**i**) The holotype from Zhejiang, (**b**,**j**) the paratype from Hunan, (**c**) the paratype from Jiangxi, (**d**,**k**) from Laos, (**e**,**l**,**p**) the paratype from Guangxi, (**f**,**m**,**q**) the paratype from Hunan, (**g**,**n**,**r**) the paratype from Zhejiang, and (**h**,**o**,**s**) from Yunnan.

**Comparative materials examined** (*C. violacea* Kerremans, 1892). 1 syntype (sex unknown) (BMNH): Haute Birmanie (Photo by Eduard Jendek, with permission, Figure 6a); 1♂: Laos, Mt. Phu Phan, alt. 2060 m, Jun 2017 (Figure 3b, Figure 4c, Figure 8c and Figure 9d,k; molecular No. LAM, failed); 1♀: China, Yunnan Province, Diqing Prefecture, Weideng Township, alt. ca 2745 m, 13–15 June 2022, leg. Xue-Zhou Li (Figure 5d, Figure 8g and Figure 9h,o,s; molecular No. YNF, failed); 2♀, Cornfield at Mopan Mountain, Xinping County, Yuxi City, Yunnan Province, China, 25 May–27 June 2023, Malaise trap, 23°58′29.08″ N, 101°56′40.50″ E, alt.1960 m, Yunnan Agricultural University; 1♂, Farmland at Sanchaha Group, Lizhi Village, Shitou Township, Yulong County, Lijiang City, Yunnan Province, China, 18 August–26 September 2023, Malaise trap, 26°47′36.32″ N, 99°38′29.25″ E, alt.2574 m, Yunnan Agricultural University (molecular No. YNM); 1♀, Yingjiang County, Dehong Dai and Jingpo Autonomous Prefecture, Yunnan Province, China, alt. 900 m, leg. Chang-gui Liu, July–August 2020, 24°38′15.61″ N, 97°50′29.75″ E; 1♀, Chayu County town, Xizang/Tibet Autonomous Region, China. 28°39′40.62″ N, 97°28′00.91″ E, alt. 2343 m, 20 July 2021, leg. Hao-Wei Liu; 1♂, 5 August 2017, Forest Park at Tuyunguan, Nanming District, Guiyang City, Guizhou Province, China, 26°33′18.94″ N, 106°45′09.50″ E, alt. 1216 m, Leg. Ming-Zhi Zhao, collected in a peach orchard using a cerambycid baited trap (new country record); and 1♀ (CHTS): Vietnam, Yên Bái Province, Yên Bái City, June 2024 (Figure 6b1–b4).

**Figure 10:**
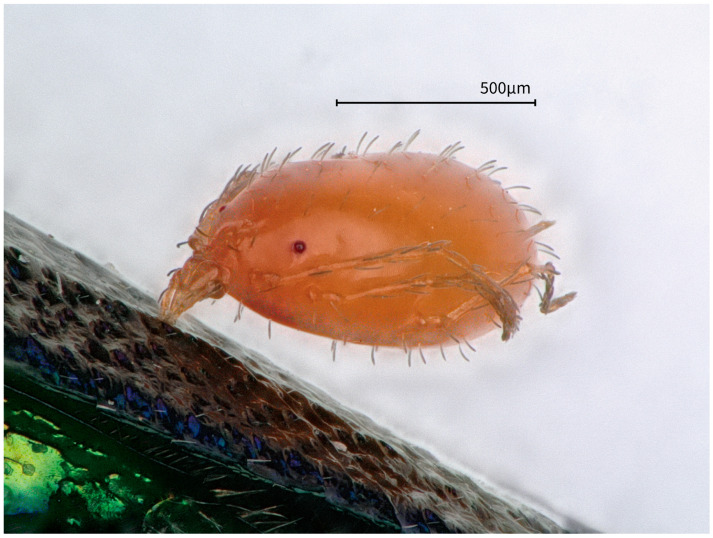
A nymphal mite (Acari: Trombidiformes, Parasitengona, cf. Erythraeidae) attached to the dorsal surface of the elytra of *Chrysobothris borealina* sp. nov. (♀). The bright orange–red larva is fixed to an exposed elytral area rather than a concealed intersegmental membrane, suggesting a transient attachment or phoretic-like position, rather than a confirmed feeding site.

### 3.3. Record of Mite Attachment on the Elytral Surface

Mite association. On the body surface of one female adult, we observed a single orange–red mite (Acari: Trombidiformes; presumably a larval individual of the family Erythraeidae) attached to the exposed dorsal surface of the elytra. No other mites were found on the remaining body surface of this specimen. Based on the 1000 µm scale bar on the photomicrograph, the body length of the mite was estimated to be approximately 0.85–0.90 mm (Figure 10).

Members of the clade Parasitengona (e.g., Erythraeidae, Trombidiidae, and related families) commonly exhibit ectoparasitic behavior on arthropods in the larval stage, with a host range that includes Coleoptera, and there are explicit records of Buprestidae as hosts. For example, larvae of *Leptus* (*Leptus*) *darvishi* have been reported as ectoparasites in beetles (Coleoptera: Buprestidae and Tenebrionidae) [22]. Likewise, records from the Iberian Peninsula indicate that *L.* (*L.*) *mirenae* parasitizes the buprestid genus *Julodis* (“parásito de *Julodis pilosa”*) [23].

However, in documented cases of true parasitic feeding, larval mites generally prefer more concealed microhabitats and tend to insert their chelicerae into softer, weakly sclerotized regions of the host cuticle. For instance, larvae of *Trombidium newelli* were recorded as occurring “beneath the elytra” and “using the chelicerae to pierce soft, weakly sclerotized regions for attachment and feeding” [24]. The range of attachment sites of phoretic mites on beetles is broader than previously assumed: the elytra can not only serve as attachment sites, but both the dorsal surface and the lateral margins of the elytra are among the common attachment areas [25]. Therefore, the condition observed in this study—only a single mite attached to an exposed dorsal elytral area—is more consistent with one of the following interpretations: (i) the mite was in a transient attachment or migratory phase (e.g., still searching for a more suitable soft-membrane feeding site), or (ii) it represented an accidental transfer or phoresy-like hitchhiking behavior, rather than evidence of established, sustained parasitic feeding, unless clear puncture marks or localized host reactions are observed at the original attachment site, or stylostome structures are recovered (which have been reported and morphologically studied in some Parasitengona) (Figure 12), and such inferences should be regarded as tentative. In addition, compiled data show that buprestids (e.g., *Agrilus biguttatus* and *A. sulcicollis*) can serve as carriers for phoretic mites, suggesting that buprestid beetles may also function as dispersal vectors for mites in ecological contexts [25].

**Figure 11:**
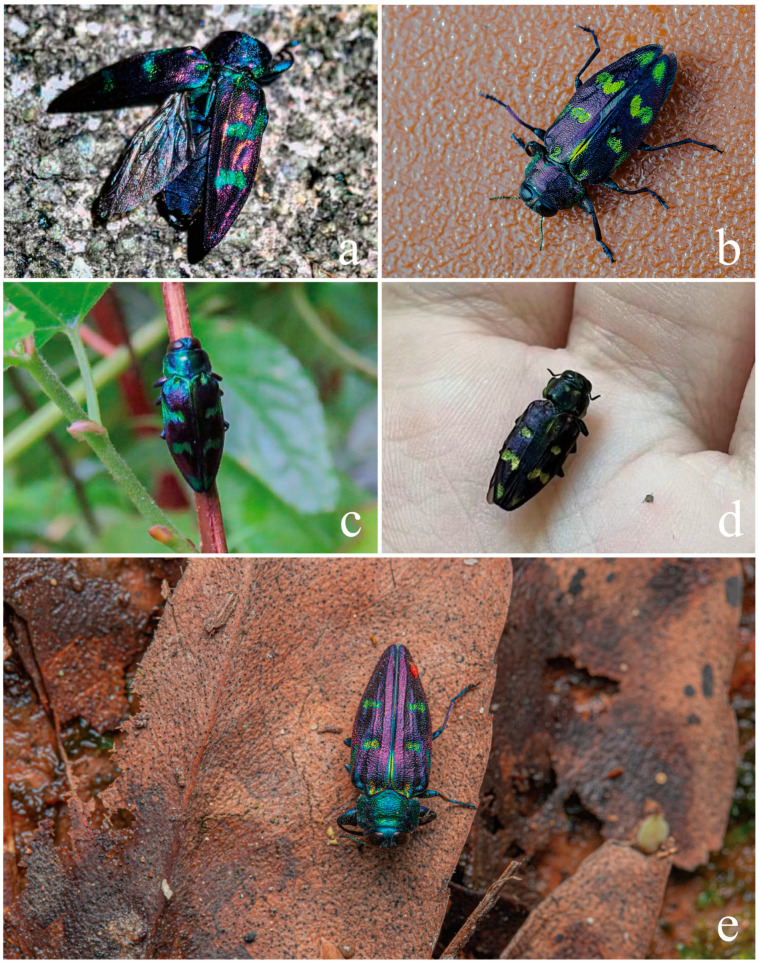
Field photographs of adults of *Chrysobothris borealina* sp. nov. in China. (**a**) Zhejiang Province, Lishui City, Longquan City, Baishanzu National Nature Reserve, by Yi-Fan Wang; (**b**) Hunan Province, Zhangjiajie City, Tianmen Mountain National Forest Park, by Ping Zhao; (**c**) Guangxi Zhuang Autonomous Region, Jinxiu Yao Autonomous County, by Jin-Teng Zhao; and (**d**) Sichuan Province, Chengdu City, Dujiangyan City, Qingchengshan Town, by Ke-Xin Hu. All images show individuals in their natural microhabitats. (**e**) Zhejiang Province, Quzhou City, Bao’an Township, Xianxialing Provincial Nature Reserve, by Xuan-Xuan He.

**Figure 12:**
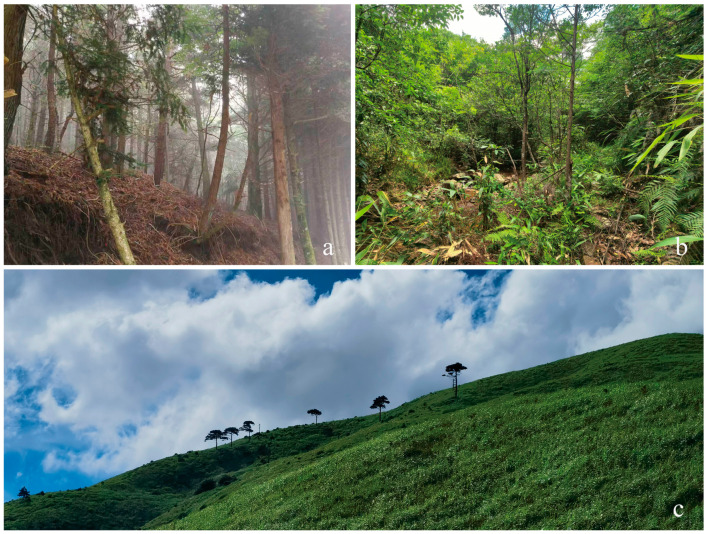
Habitats at collecting localities for *Chrysobothris borealina* sp. nov. in China. (**a**) Longquan City, Lishui, Zhejiang Province, by Yi-Fan Wang; mixed pine–broadleaf forest along a mountain road. (**b**) Bao’an Township, Xianxialing Provincial Nature Reserve, Jiangshan City, Zhejiang Province, by Xuan-Xuan He; humid montane evergreen–broadleaf forest. (**c**) Luxi County, Pingxiang City, Jiangxi Province, by Jin-Qiao Zhu; montane grassland on an exposed ridge. The photographs show the general landscape of the sites where the adults were collected.

### 3.4. Ecology

Field observations indicate that the new species is frequently encountered in environments where pine forests and their edges, mountain ravines, and ridge grasslands occur in close juxtaposition, and they are most often found in sunny forest edges or open areas, the same as most adult buprestidae [26].

**Figure 13:**
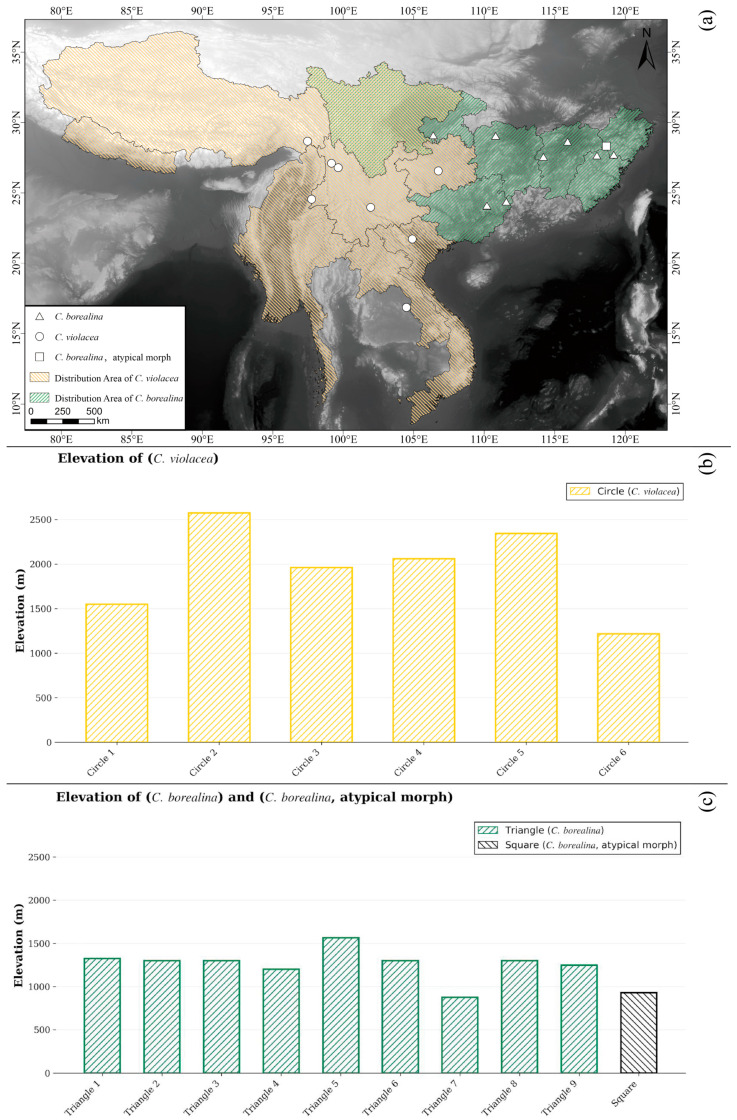
Geographic distribution (**a**) and elevational records (**b**,**c**) of *Chrysobothris borealina* sp. nov. (including an atypical morph) and *C. violacea* in China and adjacent regions.

The ecological niche of the new species of *Chrysobothris* may both differ from and overlap with that of the closely related species *C. violacea* (Figure 13). First, in altitudinal distribution, the new species was collected from 875–1566 m, whereas *C. violacea* has been recorded from 1216–2574 m**,** indicating that the latter, as a whole, inhabits higher-elevation environments. Although the altitudinal ranges of the two species overlap in the zone of approximately 1200–1500 m, the marked difference in mean elevation suggests possible ecological differentiation, i.e., partitioning of the vertical gradient by different species [27]. However, because the two species do not widely coexist in strict sympatry, their altitudinal divergence may in part reflect regional differences rather than purely competitive exclusion. The new species is distributed in eastern and southern China (Zhejiang, Jiangxi, Hunan, Chongqing, Sichuan (only by photo, but without a specimen), and Guangxi), while *C. violacea* is restricted to the montane regions of southwestern China (Sichuan, Guizhou (new province record), Yunnan, and Xizang/Tibet), Myanmar, Nepal, Laos, Vietnam, and India, so that the two species show largely separate distribution ranges with only limited potential for geographic overlap.

Additionally, the potential host plants of the new species remain speculative because oviposition or larval feeding on specific hosts has not yet been directly observed. Given that the species has so far only been found in pine forest environments, its larvae are most likely to utilize pines (*Pinus* spp.) as host plants, just like *C. cribraria* [28], rather than most species of the genus, which primarily attack broadleaved trees [29]. It is also impossible to determine whether it exhibits a taxis towards burned wood, such as the closely related genus *Phaenops* [30].

## 4. Discussion

Given the considerable phenotypic plasticity observed in *Chrysobothris*, the application of COI barcoding in this study provides a practical example of how molecular data can help delineate species boundaries within morphologically complex groups and reduce the risk of misinterpretation based on coloration and maculation patterns. During molecular analyses, DNA extraction and COI gene amplification were also attempted on two other samples of *C. violacea*, denoted as YNF (Yunnan, female) and LAM (Laos, male). However, likely due to specimen age, high-quality DNA could not be obtained. The COI gene was sequenced using two primer pairs (JERRY–PAT and LCO–HCO). Since the older specimen YNM yielded only a partial sequence with LCO–HCO, complementary data were generated using JERRY–PAT, illustrating a practical strategy for working with degraded DNA. Both datasets support our taxonomic conclusions: in both COI datasets, specimens from Zhejiang, Hunan, Guangxi and Jiangxi form a strongly supported monophyletic clade with very low intragroup K2P distances, whereas distances to *C. violacea* and other ingroup taxa are markedly higher. For both primer sets, the five samples of *C. borealina* sp. nov. consistently form a compact clade, with *C. violacea* recovered as the nearest relative among the taxa included. This is consistent with their similarity in overall habitus and several diagnostic characters.

This species adds a montane, pine-associated member of *Chrysobothris* to the fauna of South China. Its recognition relies on relatively stable external and genital characters (e.g., pronotal posterior margin, elytral sculpture and apical serration, and terminal ventrites), compared in detail with *C. violacea*. Notably, some characters that are frequently emphasized in regional keys—such as the development and serration of the tooth on the fore femur—do not contribute to separating *C. borealina* sp. nov. from *C. violacea* in our material, and thus should be treated with caution when applied to this species pair [31].

*C. borealina* shows pronounced intraspecific variation and sexual dimorphism, especially in metallic coloration and maculation; apical spots range from well-developed to nearly absent, with a uniform cupreous–violet ground color in extreme individuals. The female specimen figured as Figure 6b, which lacks typical apical elytral spots, was initially suspected to represent a distinct, closely related species. However, COI barcodes demonstrate that this specimen belongs to the same species as the remaining *C. borealina* **sp. nov.**, and its characters in elytral outline, pronotal sculpture, ovipositor, protibiae and anal ventrite also fall within the observed variation in the species.

This case highlights that, in *Chrysobothris*, metallic sheen and color pattern alone can easily lead to misinterpretation; in Buprestidae, pronounced intraspecific polymorphism and morphotypes can mask lineage diversity, and COI barcoding has repeatedly revealed cryptic or pseudocryptic lineages within nominal species complexes (e.g., *Agrilus viridis* complex; *Chrysochroa fulgidissima* complex) [2,32,33]. Additionally, reliance on external morphology alone may inflate species hypotheses in difficult groups, and integrative analyses have shown that some morphologically delimited entities can have weak or complex signals in COI-based frameworks, highlighting the need for combined evidence [34,35].

Although variation in size and color pattern has been noted in some buprestid species, detailed documentation of pronounced intraspecific variability in body size, dorsal maculation and sexual dimorphism remains relatively rare in *Chrysobothris*. Most species are considered morphologically uniform, with only limited remarks on variability such as in a few North American taxa [10,36,37].

The delimitation of new species should prioritize structural characters, especially those of the male genitalia, and, whenever possible, be supported by molecular evidence to avoid oversplitting highly variable taxa. Similar integrative approaches combining morphology and DNA barcoding have proved particularly effective for wood-boring beetles (Cerambycidae and Buprestidae), enhancing species-level identifications and revealing cryptic diversity within morphologically complex groups [38,39,40]. More broadly, integrative taxonomy that combines multiple and complementary data sources is now widely established as best practice for robust species delimitation in insects [11,41,42].

From a phytosanitary and applied standpoint in China, accurate identification of *Chrysobothris* species is of growing importance, as buprestid borers are repeatedly intercepted in international timber trade and wood-packaging pathways. For example, *Chrysobothris igniventris* Reitter, 1895, an occasionally intercepted buprestid, was reported during inspection procedures, with its bio-geographic origin, interception status, and larval host associations (including *Larix* and *Pinus*) summarized for pest risk assessment [43]. Interception-based taxonomic studies have further documented the occurrence of *Chrysobothris* species recovered from wooden packaging imported from the United States, emphasizing the practical necessity of distinguishing morphologically similar species [44]. More broadly, multiple buprestid genera, including *Chrysobothris*, have been intercepted on timbers from Papua New Guinea, underscoring the diversity of wood-boring beetles potentially transported through global trade networks [45]. Although the larval host of *C. borealina* remains undetermined, targeted rearing efforts or systematic gallery surveys within pine-associated habitats could help clarify its potential relevance to forestry.

## Figures and Tables

**Table 1:** PCR primers used for amplification of mitochondrial COI in this study, with their sequences, references and sample usage.

Primer Name	Primer Sequence (5′-3′)	Citation	Usage
LCO1490	GGTCAACAAATCATAAAGATATTGG	[20]	PCR for samples ZJM, GXF01, HNF, ZJF, and HNM
HCO2198	TAAACTTCAGGGTGACCAAAAAATCA		
JERRY	CAACATTTATTTTGATTTTTTGG	[21]	PCR for samples YNM, GXF02, JXM, HNF, and ZJF
PAT	TCATTGCACTAATCTGCCATATTA		

Sample codes are structured as follows: the first two letters represent the Chinese province (ZJ for Zhejiang; GX for Guangxi; HN for Hunan; YN for Yunnan; and JX for Jiangxi) or Laos (LA), the third letter denotes sex (M for male and F for female), and the subsequent numerals designate individual specimens. The code YNM corresponds to “*Chrysobothris violacea*”. This nomenclature convention is consistently applied throughout the subsequent tables and figures.

**Table 2:** Kimura 2-parameter genetic distances (K-2P) of mitochondrial COI sequences among samples (ZJM, GXF01, HNF, ZJF, and HNM, later as *Chrysobothris borealina* sp. nov.) and selected related taxa based on the LCO–HCO primer group.

	1	2	3	4	5	6	7	8	9	10	11	12	13	14	15
1		0.005	0.004	0.004	0.004	0.091	0.081	0.097	0.091	0.128	0.092	0.089	0.107	0.097	0.102
2	0.018		0.004	0.005	0.004	0.088	0.077	0.096	0.087	0.120	0.088	0.082	0.100	0.092	0.098
3	0.013	0.011		0.005	0.000	0.087	0.077	0.095	0.087	0.125	0.087	0.085	0.101	0.093	0.101
4	0.013	0.018	0.016		0.005	0.094	0.080	0.094	0.089	0.127	0.090	0.083	0.103	0.097	0.099
5	0.013	0.011	0.000	0.016		0.087	0.077	0.095	0.087	0.125	0.087	0.085	0.101	0.093	0.101
6	1.085	1.060	1.060	1.096	1.060		0.013	0.018	0.017	0.019	0.017	0.018	0.017	0.017	0.020
7	1.022	0.993	1.000	1.016	1.000	0.105		0.016	0.016	0.018	0.016	0.017	0.017	0.016	0.018
8	1.111	1.102	1.102	1.096	1.102	0.183	0.176		0.018	0.019	0.019	0.018	0.017	0.019	0.020
9	1.094	1.069	1.069	1.087	1.069	0.160	0.158	0.185		0.020	0.016	0.018	0.019	0.019	0.020
10	1.226	1.208	1.230	1.234	1.230	0.199	0.202	0.202	0.225		0.018	0.021	0.019	0.020	0.020
11	1.072	1.053	1.053	1.062	1.053	0.160	0.154	0.212	0.160	0.202		0.014	0.017	0.020	0.020
12	1.071	1.037	1.052	1.046	1.052	0.184	0.162	0.184	0.175	0.234	0.118		0.019	0.018	0.021
13	1.154	1.120	1.130	1.142	1.130	0.172	0.168	0.179	0.204	0.203	0.184	0.196		0.021	0.020
14	1.102	1.077	1.085	1.104	1.085	0.182	0.157	0.221	0.196	0.235	0.200	0.196	0.209		0.020
15	1.128	1.110	1.128	1.112	1.128	0.224	0.200	0.224	0.222	0.231	0.230	0.237	0.215	0.215	

1. ZJM, 2. GXF01, 3. HNF, 4. ZJF, 5. HNM, 6. DQ222000 Chrysobothris affinis, 7. KM364359 Chrysobothris merkelii, 8. NC012765 Chrysochroa fulgidissima, 9. NC057197 Melanophila acuminata, 10. NC062816 Chalcophora japonica, 11. NC063147 Coomaniella dentata, 12. NC063146 Coomaniella copipes, 13. NC063127 Dicerca corrugata, 14. NC081980 Agrilus zanthoxylumi, 15. NC064400 Agrilus ornatus.

**Table 3:** Kimura 2-parameter genetic distances (K-2P) of mitochondrial COI sequences among samples (HNF, ZJF, GXF02, and JXM, later as *Chrysobothris borealina* sp. nov.), *C. violacea* (YNM), and selected Buprestidae, based on the JERRY–PAT primer group.

	1	2	3	4	5	6	7	8	9	10	11	12	13	14	15
1		0.004	0.004	0.004	0.010	0.012	0.013	0.016	0.014	0.017	0.016	0.017	0.016	0.016	0.018
2	0.014		0.004	0.004	0.009	0.013	0.013	0.016	0.014	0.017	0.016	0.017	0.017	0.017	0.018
3	0.016	0.010		0.004	0.009	0.012	0.012	0.016	0.014	0.017	0.016	0.017	0.016	0.017	0.017
4	0.013	0.010	0.010		0.009	0.012	0.012	0.017	0.014	0.017	0.016	0.017	0.017	0.016	0.018
5	0.075	0.071	0.071	0.067		0.013	0.013	0.017	0.014	0.017	0.016	0.017	0.016	0.016	0.018
6	0.124	0.123	0.121	0.121	0.135		0.013	0.017	0.015	0.018	0.017	0.019	0.016	0.017	0.020
7	0.122	0.121	0.116	0.118	0.127	0.116		0.016	0.015	0.017	0.017	0.017	0.017	0.016	0.019
8	0.186	0.186	0.188	0.188	0.199	0.200	0.187		0.016	0.018	0.019	0.016	0.017	0.019	0.019
9	0.154	0.159	0.152	0.159	0.167	0.167	0.163	0.198		0.018	0.015	0.016	0.017	0.018	0.018
10	0.220	0.231	0.223	0.220	0.222	0.224	0.226	0.214	0.241		0.019	0.020	0.020	0.020	0.021
11	0.188	0.191	0.188	0.193	0.195	0.180	0.180	0.232	0.181	0.230		0.013	0.017	0.018	0.021
12	0.183	0.183	0.183	0.185	0.183	0.200	0.178	0.195	0.184	0.251	0.120		0.017	0.019	0.020
13	0.211	0.209	0.209	0.209	0.200	0.194	0.209	0.205	0.223	0.228	0.207	0.221		0.019	0.019
14	0.193	0.203	0.198	0.198	0.188	0.203	0.196	0.254	0.221	0.258	0.222	0.226	0.237		0.019
15	0.227	0.225	0.223	0.232	0.231	0.262	0.242	0.263	0.249	0.256	0.263	0.258	0.247	0.225	

1. HNF, 2. ZJF, 3. GXF02, 4. JXM, 5. YNM, 6. DQ222000 *Chrysobothris affinis*, 7. KM364359 *Chrysobothris merkelii*, 8. NC012765 *Chrysochroa fulgidissima*, 9. NC057197 *Melanophila acuminata*, 10. NC062816 *Chalcophora japonica*, 11. NC063147 *Coomaniella dentata*, 12. NC063146 *Coomaniella copipes*, 13. NC063127 *Dicerca corrugata*, 14. NC081980 *Agrilus zanthoxylumi*, 15. NC064400 *Agrilus ornatus.*

**Table 4:** Differential diagnosis between *Chrysobothris borealina* **sp. nov.** and *C. violacea* Kerremans, 1892.

Character	*C. borealina* sp. nov.	*C. violacea* Kerremans, 1892
**Body** **shape**	Body distinctly shorter and wider. Elytral lateral margins slightly diverge from the base to midlength (or slightly beyond), then sharply attenuate toward apices. Females clearly larger than males, but overall more slender in habitus (Figure 8). From the midlength rearwards, the lateral margins are nearly straight and converge posteriorly.	Body more elongate and broadly oval. Elytral lateral margins subparallel in the anterior two thirds, then gradually converge toward the apices. Lateral margins with an arcuate outline, gradually narrowing toward apical portions of elytra and ventrites.
**Coloration**	Ground color dark purple to purplish–blue with strong metallic luster. Frons and pronotal margins often with extensive bluish–green to golden–green reflections. Elytral maculation predominantly golden–green, commonly forming transverse bands or vermiform markings. Maculae vary markedly in size, shape, and extent, producing a dynamic, aurora-like pattern (Figure 5a–c and Figure 3a,c), usually with distinct golden–green apical macula reaching elytral margin. Legs mostly bluish–green to bluish–violet.	Overall coloration darker, ranging from purplish–brown to dark copper–brown. Yellowish–green reflections weak and are mostly restricted to the inner margins of the compound eyes, the anterior part of the frons, and the narrow bands along the pronotal margins. Elytral maculation mainly consists of small yellow to golden yellow oval spots (sometimes forming small transverse extensions at midlength, or appearing pale yellowish–green (Figure 6a)), which are scattered and do not form large patches or conspicuous apical maculae. Legs more dark purplish–brown.
**Pronotum**	Median lobe on posterior margin shorter and broader, with a blunt apex; posterior margin outline appearing more nearly straight and broadly rounded. Apex of median lobe rounded and subtruncate.	Median lobe on posterior margin longer, narrower, and more acute, giving the posterior margin a more pointed appearance. Apex of median lobe angulate.
**Clypeus**	Anterior margin of clypeus distinctly and deeply bisinuate; median elevation relatively narrow and somewhat prominent, see Figure 3a1,c1.	Anterior margin of clypeus only shallowly bisinuate; median elevation broader, more obtuse, and less prominent, see Figure 3b1.
**Elytra**	Elytral punctures distinctly coarser and deeper, surface appearing somewhat roughened, see Figure 3a2,c2. Apicolateral margin with closely set, regular small teeth, and slightly narrower and more acute apices. Subapical lateral patch with distinct medium-sized punctures, surface rugose, Figure 3a3,c3.	Elytral punctures finer, denser, and shallower, surface appearing smoother and more polished, see Figure 3b2. Apicolateral margin with larger but more sparsely arranged teeth, with apices broader and more rounded. Subapical lateral patch almost impunctate, surface relatively smooth, Figure 3b3.
**Abdominal Ventrite**	Male anal ventrite with a broad, shallow V-shaped apical emargination and rather obtuse lateral angles; disc coarsely and densely punctate, without a distinct smooth or sparsely punctate median stripe. Female anal ventrite relatively broad, with only a shallow apical emargination and somewhat coarser punctation, See Figure 3a4,c4.	Male anal ventrite with a distinctly deeper and narrower V-shaped apical emargination; disc bearing a well-defined smooth or sparsely punctate median stripe, the surrounding punctation being finer and more evenly distributed. Female anal ventrite distinctly narrower and nearly semicircular in outline, with a deeper apical emargination and finer, more evenly distributed punctation, see Figure 3b4.
**Aedeagus/Genitalia**	In dorsal view, the median lobe, as shown in Figure 4a,b, bears a continuous row of small denticles, giving its outline a distinctly finely serrate appearance. The outer margin of the parameres is distinctly arcuate from midlength and then abruptly converges towards the apex, forming a narrow, sickle-shaped outline with strong curvature.	The outer margin of the median lobe, at the position indicated by the blue arrow in Figure 4c, is completely smooth and unarmed in dorsal view. In ventral view, the parameres are stouter, with the outer margin only slightly arcuate and the overall outline more gently curved.
**Distribution**	China: Zhejiang, Hunan, Jiangxi, Fujian, Guangxi, Chongqing, and Sichuan (only by photo).	China: Yunnan, Sichuan, Xizang (Tibet), Guizhou (new province record); Laos, Myanmar, Nepal, Vietnam, and India.

## Data Availability

The original contributions presented in this study are included in the article. Further inquiries can be directed to the corresponding authors.
